# High willingness to use overdose prevention sites among suburban people who use drugs who do not inject

**DOI:** 10.1186/s12954-023-00865-z

**Published:** 2023-09-21

**Authors:** Kristin E. Schneider, Glenna J. Urquhart, Saba Rouhani, Sean T. Allen, Miles Morris, Susan G. Sherman

**Affiliations:** 1 Department of Health, Behavior and Society, Johns Hopkins Bloomberg School of Public Health, 624 N. Broadway, HH186, Baltimore, MD 21205 USA; 2 Department of Epidemiology, New York University School of Global Public Health, 708 Broadway, New York, NY 10003 USA

**Keywords:** Overdose prevention sites, Non-injection drug use, Harm reduction, PWUD, Suburban

## Abstract

**Introduction:**

Overdose prevention sites (OPS) are evidence-based interventions to improve public health, yet implementation has been limited in the USA due to a variety of legal impediments. Studies in various US settings have shown a high willingness to use OPS among urban and rural people who inject drugs, but data among people who use drugs (PWUD) via non-injection routes of administration in suburban areas are lacking.

**Methods:**

We utilized cross-sectional data from a sample of suburban PWUD who have not injected drugs in the past 3 months (*N* = 126) in Anne Arundel County, Maryland. We assessed PWUDs’ likelihood of using a hypothetical OPS and perceived potential barriers to accessing OPS. We tested for associations between sociodemographic characteristics, drug use, service access, and overdose experiences with willingness to utilize OPS.

**Findings:**

Participants’ median age was 42, and the majority were men (67%) and non-Hispanic Black (79%). Sixty-six percent reported willingness to use an OPS. Concerns about confidentiality (29%), arrest (20%), and transportation costs (22%) were the most anticipated barriers to using OPS. Men (75% vs 55%, *p* = 0.015), participants who used heroin (53% vs 32%, *p* = 0.017), and participants who used multiple overdose prevention behaviors (e.g., using fentanyl test strips) (36% vs 19%, *p* = 0.006) were more likely to report willingness to use OPS.

**Conclusion:**

Most suburban non-injecting PWUD in the sample were willing to use an OPS. OPS implementation strategies in suburban settings should be tailored to reach PWUD via non-injection routes of administration while meeting the unique needs of suburban contexts.

## Introduction

Overdose rates surged during the COVID-19 pandemic; a record-breaking 100,000 people died from drug overdoses between April 2020 and April 2021 in the USA, a nearly 30% increase from the prior 12-month period [1]. This persisting crisis is shaped by a number of intersecting socio-structural factors that increase likelihood of overdose including changes in the drug markets (e.g., the pervasiveness of fentanyl) [2, 3], impacts of the COVID-19 pandemic (e.g., harm reduction and drug treatment services interruptions), structural vulnerabilities (e.g., housing and food insecurity) experienced by people who use drugs (PWUD) [4], and drug criminalization [5, 6]. PWUD lacking safe, private locations may rush drug use to avoid police encounters, increasing the likelihood of overdose. Others hesitate to or completely avoid contacting emergency services during overdoses for fear of arrest, making some overdoses more likely to be fatal [7, 8]. Long-term reductions in overdoses require harm reduction interventions, developed in partnership with PWUD, that address social and structural drivers of overdose mortality [9].

Overdose prevention sites (OPS), sometimes referred to as supervised injection facilities or safe consumption sites, are evidence-based interventions implemented throughout Europe, Australia, and Canada that can fundamentally alter this risk environment [10]. OPS allow individuals to use previously acquired drugs under the supervision of medical personnel trained to reverse overdoses, provide harm reduction counseling, case management, and link PWUD to resources, such as primary care and drug treatment [10, 11]. Studies of OPS demonstrate reductions in overdose, infectious disease incidence, improper syringe disposal, and crime [12–17]. Studies have documented high willingness to use OPS among PWUD in several locations throughout the U.S [18–23]. Existing evidence on OPS willingness in the USA has focused on urban settings and people who inject drugs rather than people who use drugs via other routes of administration (e.g., smoking, snorting). Little is known about willingness to use OPS among suburban PWUD and among PWUD who do not inject. In this study, we asked a sample of PWUD who do not inject to about their willingness and potential barriers to utilizing an OPS in Anne Arundel County, Maryland.

## Methods

### Study design

We used data from the Peer harm Reduction Of Maryland Outreach Tiered Evaluation (PROMOTE), a mixed-methods, cross-sectional study of people who use drugs (PWUD) in Baltimore City and Anne Arundel County (AAC), Maryland. Adjacent to Baltimore City, AAC has the third-most unintentional overdose deaths in the state and is largely constituted of suburban (more sprawling communities with limited access to public transportation) communities with limited harm reduction service coverage [24]. In AAC, PWUD were recruited from seven street-based locations between November 2019 and March 2020; data collection ended prematurely due to the COVID-19 pandemic. We created heat maps of areas with high drug activity using drug arrest and overdose data from the Anne Arundel County and Annapolis City police departments. We extracted time signatures within these areas to develop a time–day–location sampling frame. Based on the time signatures, the study team created monthly recruitment schedules and parked the study van in seven recruitment zones. Once the study team reached a recruitment zone, they recruited participants on foot or by talking to interested people who came by a table with harm reduction supplies setup outside the van. All study procedures occurred inside the study van to ensure confidentiality and privacy. Eligibility criteria for the AAC surveys required participants to report being at least 18 years old and non-prescription use of any opioid in the previous 6 months. Eligible participants provided informed consent and completed a 30-min Audio Computer-Assisted Self-Interview (ACASI) on a tablet with a trained interviewer present for technical assistance. Following the survey, respondents were compensated with a $25 USD VISA gift card. In total, 173 participants were recruited. This analysis is restricted to participants who did not report injection drug use in the past 3 months (*n* = 132) with complete data for the outcome measure (*N* = 126). This study was approved by the Johns Hopkins Bloomberg School of Public Health and the Maryland Department of Health Institutional Review Boards.

### Measures

#### Willingness to use OPS

Each participant received the following description of OPS: “*An overdose prevention site, also known as a safe consumption space, is a place where it would be legal for people to safely inject, snort, or smoke, or otherwise consume drugs that they buy somewhere else. You would not be arrested while in the site. There would be staff on site to respond to an overdose, and to provide basic medical care and referrals to health and social services upon request. While overdose prevention sites are not currently approved in the USA, they operate in several countries worldwide.*” Participants then indicated their likelihood of using OPS by selecting their response from a Likert scale (very likely, somewhat likely, somewhat unlikely, very unlikely), which was dichotomized for analysis (likely vs. unlikely), consistent with prior work [21].

#### Awareness of and barriers to using OPS

After receiving a description of OPS, participants shared whether they had previously heard of OPS (yes/no). Participants were asked to select anticipated barriers to OPS access (yes/no) from a predetermined list of 10 options, including concerns about arrest, confidentiality, cost of transportation, and lack of interest. We summed endorsement of these 10 barriers to create a categorical variable for analysis (number of anticipated barriers: 0, 1, 2 +).

#### Sociodemographic characteristics

Sociodemographic characteristics included age (continuous), gender (man/woman; no participants reported non-binary gender identities), race/ethnicity (trichotomized: non-Hispanic white, non-Hispanic Black, other races (including Hispanic, Asian, Native American, and Multiracial), and educational attainment (dichotomized: high school diploma or G.E.D. vs. did not graduate high school). Structural vulnerabilities included currently experiencing homelessness (yes/no), recently experiencing food insecurity (dichotomized: weekly or more compared to less than weekly in the past 3 months) and arrest within the past year (yes/no).

#### Drug use characteristics

Participants were asked about their recent (past 3 months) use of eight substances (i.e., opioid pills, heroin, fentanyl, cocaine, crack, benzodiazepines/tranquilizers, synthetic cannabinoids, and PCP) and method of use (i.e., smoking, snorting, swallowing). We generated binary variables to indicate any recent non-injection use of each drug and created a dichotomous variable to identify participants who had recently used three or more substances vs. less. Lifetime history of any drug injection was captured as a binary variable (yes/no). Participants selected their typical locations for drug use from a predetermined list including: your or someone else’s home, street/park, abandoned building, shooting gallery, car/vehicle/bus/metro, stairwells, public bathroom, other. Other locations were then specified by participants using free text. We then created a categorical variable for public/semi-public (e.g., shooting galleries, abandoned buildings, streets, or parks) and private locations (e.g., at home or at someone else’s home), consistent with our prior work [7].

#### Overdose experiences and prevention behaviors

Lifetime and past 6-month overdose experiences were assessed with binary variables (yes/no). Participants were also asked what they do to prevent overdose (yes/no) from a list of six behaviors: buying from the same dealer; using fentanyl test strips; keeping naloxone with me; using with other people; calling or texting a friend to let them know what I’m doing; and using in a place where someone will see me if I overdose. We summed endorsement of these items to generate a categorical variable (number of overdose prevention behaviors: 0, 1, 2 +).

### Analysis

Covariates of interest were selected a priori based on prior work and hypothesized associations between covariates and willingness to use OPS. We compared differences in covariates of interest by OPS likelihood using Chi-squared tests for categorical variables with cell sizes > 10, Fisher’s exact tests for categorical variables with cell sizes ≤ 10, and two-sample Mann–Whitney tests for continuous variables. Descriptive analyses were conducted in Stata SE 15.1.

## Results

Participants’ median age was 42 years and most reported being men (66%) and non-Hispanic Black (79%; Table 1). Sixty-five percent of participants had received a high school diploma or equivalent. Many participants reported experiencing structural vulnerabilities, as half (52%) were currently unhoused and 39% experienced recent food insecurity. One-third (31%) had been arrested in the past year. Most participants had never injected drugs (89%). The most prevalent non-injection drugs recently reported included opioid pills (66%), crack (53%), heroin (44%), fentanyl (42%), and cocaine (41%). Multiple substance use was common, with 64% reporting recent use of three or more drugs. Thirty-five percent of participants reported having ever overdosed, and 14% reported having an overdose in the previous 6 months. Participants reported implementing several overdose prevention behaviors. The most common strategies included buying from the same dealer (29%), using in a visible location (25%), and using fentanyl test strips (18%). Forty-four percent had heard of OPS before it was described to them, and 58% reported being likely to use OPS if available (37% very likely, 21% somewhat likely, 9% somewhat unlikely, 33% very unlikely). The most common perceived barriers to using OPS were concerns about confidentiality (29%), cost of transportation (22%), and concerns about arrest (20%).

**Table 1:** Factors associated with OPS willingness among PWUD who do not inject in Anne Arundel County, Maryland, 2019–2020 (*N* = 126)

	Willing to use OPS			
	Total	No	Yes	*p*
	*N* = 126	53 (42.1)	73 (57.9)	
**Sociodemographic characteristics**				
Age (median, IQR)	42 (20)	41 (21)	43 (18)	0.778^a^
Gender				
Woman	43 (34.1)	24 (45.3)	19 (26.0)	**0.024**
Man	83 (65.9)	29 (54.7)	54 (74.0)	
Race				
Non-Hispanic white	16 (13.1)	7 (13.7)	9 (12.7)	0.893^b^
Non-Hispanic Black	96 (78.7)	39 (76.5)	57 (80.3)	
Other races	10 (8.2)	5 (9.8)	5 (7.0)	
High school equivalent education	81 (64.8)	36 (67.9)	45 (62.5)	0.530
Homeless, currently	66 (52.4)	27 (50.9)	39 (53.4)	0.783
Weekly food insecurity, past 3 months	49 (38.9)	18 (34.0)	31 (42.5)	0.334
Arrested, past year	39 (31.0)	13 (24.5)	26 (35.6)	0.184
**Drug use**				
Injection drug use, ever	14 (11.1)	7 (13.2)	7 (9.6)	0.574^b^
Non-injection drug use, past 3 months				
Opioid pills	83 (66.4)	35 (67.3)	48 (65.8)	0.856
Heroin	56 (44.4)	17 (32.1)	39 (53.4)	**0.017**
Fentanyl	53 (42.1)	17 (32.1)	36 (49.3)	0.053
Crack	67 (53.2)	28 (52.8)	39 (53.4)	0.947
Cocaine	52 (41.3)	23 (43.4)	29 (39.7)	0.680
Synthetic cannabinoids	33 (26.2)	13 (24.5)	20 (27.4)	0.718
Tranquilizers	41 (32.8)	20 (38.5)	21 (28.8)	0.255
PCP	31 (24.6)	16 (30.2)	15 (20.6)	0.215
Used 3 or more substances, past 3 months	80 (63.5)	29 (54.7)	51 (69.9)	0.081
Public/semi-public drug use	78 (61.9)	29 (54.7)	49 (67.1)	0.157
**Overdose experiences and prevention**				
Ever overdosed	44 (34.9)	15 (28.3)	29 (39.7)	0.184
Overdosed in past 6 months	17 (13.6)	6 (11.5)	11 (15.1)	0.609^b^
Overdose prevention behaviors				
Buy from the same dealer	35 (29.2)	8 (16.3)	27 (38.0)	**0.014**^**b**^
Use fentanyl test strips	22 (18.3)	4 (8.2)	18 (25.4)	**0.018**^**b**^
Keep naloxone with me	21 (17.5)	6 (12.2)	15 (21.1)	0.232^b^
Use with other people	20 (16.7)	9 (18.4)	11 (15.5)	0.804^b^
Call or text a friend to let them know what I'm doing	15 (12.5)	5 (10.2)	10 (14.1)	0.587^b^
Use in a place where someone will see me if I overdose	30 (25.0)	9 (18.4)	21 (29.6)	0.201^b^
Number of overdose prevention behaviors				
0	38 (30.2)	22 (41.5)	16 (21.9)	**0.006**^**b**^
1	58 (46.0)	25 (47.2)	33 (45.2)	
2 +	30 (23.8)	6 (11.3)	24 (32.9)	
**OPS awareness and anticipated barriers**				
Ever heard of OPS	56 (44.4)	20 (37.7)	36 (49.3)	0.197
Anticipated barriers to OPS				
Concerns about arrest	24 (20.2)	6 (12.2)	18 (25.7)	0.103^b^
Confidentiality	35 (29.4)	10 (20.4)	25 (35.7)	0.101^b^
Prefer more privacy	20 (16.8)	6 (12.2)	14 (20.0)	0.325^b^
Cost of transportation	26 (21.8)	6 (12.2)	20 (28.6)	**0.043**^**b**^
Time to get there/too far	16 (13.4)	3 (6.1)	13 (18.6)	0.059^b^
Disliking the staff	6 (5.0)	1 (2.0)	5 (7.1)	0.399^b^
Childcare barriers	11 (9.2)	5 (10.2)	6 (8.6)	0.759^b^
Work/school	6 (5.0)	0 (0.0)	6 (8.6)	**–**
Illness	9 (7.6)	2 (4.1)	7 (10.0)	0.304^b^
Lack of interest	16 (13.4)	7 (14.3)	9 (12.9)	1.000^b^
Number of anticipated barriers to OPS				
0	52 (41.3)	28 (52.8)	24 (32.9)	**0.048**^**b**^
1	38 (30.2)	15 (28.3)	23 (31.5)	
2 +	36 (28.6)	10 (18.9)	26 (35.6)	

^a^Two-sample Mann–Whitney test

^b^Fisher's exact test

–Significance tests are not presented due to zero cells

Participants willing to use OPS were significantly more likely to be men (74% vs 55%, *p* = 0.024) and to have recently used heroin (53% vs 32%, *p* = 0.017) than those unwilling to use OPS. Those more willing to use OPS also reported engaging in a significantly greater (2 or more) number of overdose prevention behaviors compared to those who were unwilling (33% vs. 11%, *p* = 0.006). Overdose prevention behaviors were significantly more common among those willing to use OPS than those who were not, including buying from the same dealer (38% vs. 16%, *p* = 0.014) and using fentanyl test strips (25% vs. 8%, *p* = 0.018). Those willing to use OPS anticipated a significantly greater number of barriers (2 or more) to OPS utilization compared to those who were unwilling (36% vs. 19%, *p* = 0.048).

## Discussion

We found high willingness to use OPS among a sample of suburban PWUD who did not inject. Our finding adds to the growing evidence showing high OPS acceptability among people who inject drugs in urban and rural areas [18, 21, 23]. Individuals who use drugs but do not inject are seldom included in discussions of OPS. Our findings speak to the demand for OPS among suburban people who smoke, snort, or swallow their drugs, highlighting the importance of expanding OPS framing to consider diverse PWUD beyond urban areas.

Two-thirds of participants had previously overdosed and most engaged in behaviors associated with increased overdose risk, including public/semi-public [7] and polysubstance drug use [2]. Consistent with prior work, heroin use was associated with OPS willingness [20, 21]. We previously documented limited awareness of naloxone access points and limited knowledge of legal protections for help-seeking during an overdose in this population [8]. These findings demonstrate how PWUD but do not inject are at an increased risk for overdose because of social and structural marginalization. Participants who were likely to use OPS reported implementing overdose prevention behaviors, including using fentanyl test strips and buying from the same dealer. Participants also reported engagement with social supports to reduce overdose risk, including using around others or while on the phone with a friend. These findings demonstrate how PWUD without access to OPS are mobilizing to prevent overdoses, and structural interventions, like OPS, are needed to ensure ongoing access to harm reduction services to mitigate overdose risk.

Many PWUD anticipated barriers to accessing an OPS, and anticipated barriers were more common among those willing to an OPS than those who were not. Commonly reported concerns included transportation cost, confidentiality, and arrest, consistent with previous research in urban settings [18, 21]. However, approaches to eliminating these barriers should be tailored to meet needs of PWUD in suburban environments, where there may be greater travel barriers (farther distances to navigate and lack of public transport), less population density, and variation in PWUD/law enforcement relationships. Overcoming transportation barriers for suburban and rural PWUD requires structural approaches that can benefit health service access beyond just OPS access. Vouchers for taxis, dedicated shuttle services, and improving public health infrastructure all have a role in overcoming this key barrier. Input from local constituents—including PWUD who do and do not inject—should be at the forefront in shaping decisions around OPS implementation.

### Limitations

Data collection was stopped prematurely due to the COVID-19 pandemic, resulting in a modest sample size that limited our ability to explore additional correlates or perform adjusted analyses. The survey also did not capture methamphetamine use or differentiate between overdoses caused by opioids versus other drugs. It is also possible that participants did not fully understand the description of the OPS presented in the survey, which may have influenced their responses to questions about willingness to use an OPS or any barriers. It is also possible that relevant barriers to PWUD were not included in the survey question and are therefore missing from this analysis. Participants may have also had different levels of previous awareness of OPS that were not captured in our measures that could have influenced their perceptions of barriers or willingness to use an OPS. Future qualitative work is warranted to further explore and verify our interpretations around this topic. Additionally, our findings reflect PWUD in suburban Maryland; demand for OPS and anticipated barriers to access may differ for people in other settings.

### Conclusions

We found high OPS willingness among suburban PWUD who do not inject, extending the growing evidence of high demand for OPS throughout the USA among diverse populations of PWUD. Participants anticipated several barriers to OPS access. While many of these barriers are consistent with existing literature, experiences of these barriers may differ between PWUD who do and do not inject in urban, suburban, and rural settings. OPS expansion in the USA should facilitate access for diverse PWUD and tailor strategies to specific environments and structural contexts.

## References

[1] Ahmad F, Rossen L, Sutton P. Vital statistics rapid release - provisional drug overdose Data. 2021. https://www.cdc.gov/nchs/nvss/vsrr/drug-overdose-data.htm#citation. Accessed December 21, 2021.

[2] Binswanger IA, Stern MF, Deyo RA (2007). Release from prison–a high risk of death for former inmates. N Engl J Med.

[3] Cooper H (2015). War on drugs policing and police brutality. Subst Use Misuse.

[4] Hunter K, Park JN, Allen ST (2018). Safe and unsafe spaces: Non-fatal overdose, arrest, and receptive syringe sharing among people who inject drugs in public and semi-public spaces in Baltimore City. Int J Drug Policy.

[5] Rouhani S, Schneider KE, Rao A (2021). Perceived vulnerability to overdose-related arrests among people who use drugs in Maryland. Int J Drug Policy..

[6] Park JN, Rouhani S, Beletsky L, Vincent L, Saloner B, Sherman SG (2020). Situating the continuum of overdose risk in the social determinants of health: a new conceptual framework. Milbank Q.

[7] Potier C, Laprévote V, Dubois-Arber F, Cottencin O, Rolland B (2014). Supervised injection services: What has been demonstrated? A systematic literature review. Drug Alcohol Depend.

[8] Kerman N, Manoni-Millar S, Cormier L, Cahill T, Sylvestre J (2020). “It’s not just injecting drugs”: Supervised consumption sites and the social determinants of health. Drug Alcohol Depend..

[9] Wood E, Tyndall MW, Montaner JS, Kerr T (2006). Summary of findings from the evaluation of a pilot medically supervised safer injecting facility. CMAJ.

[10] Salmon AM, Dwyer R, Jauncey M, van Beek I, Topp L, Maher L (2009). Injecting-related injury and disease among clients of a supervised injecting facility. Drug Alcohol Depend.

[11] Kral AH, Davidson PJ (2017). Addressing the Nation’s opioid epidemic: lessons from an unsanctioned supervised injection site in the U.S. Am J Prev Med..

[12] Davidson PJ, Lambdin BH, Browne EN, Wenger LD, Kral AH (2021). Impact of an unsanctioned safe consumption site on criminal activity, 2010–2019. Drug Alcohol Depend..

[13] Green TC, Hankins CA, Palmer D, Boivin J-F, Platt R (2004). My place, your place, or a safer place. Can J Public Heal.

[14] Kral AH, Lambdin BH, Wenger LD, Davidson PJ (2020). Evaluation of an unsanctioned safe consumption site in the United States. N Engl J Med.

[15] Park JN, Sherman SG, Rouhani S (2019). Willingness to use safe consumption spaces among opioid users at high risk of fentanyl overdose in Baltimore, Providence, and Boston. J Urban Heal.

[16] Harris RE, Richardson J, Frasso R, Anderson ED (2018). Perceptions about supervised injection facilities among people who inject drugs in Philadelphia. Int J Drug Policy.

[17] Bouvier BA, Elston B, Hadland SE, Green TC, Marshall BDL (2017). Willingness to use a supervised injection facility among young adults who use prescription opioids non-medically: a cross-sectional study. Harm Reduct J.

[18] Rouhani S, White RH, Park JN, Sherman SG (2020). High willingness to use overdose prevention sites among female sex workers in Baltimore Maryland. Drug Alcohol Depend..

[19] Kral AH, Wenger L, Carpenter L, Wood E, Kerr T, Bourgois P (2010). Acceptability of a safer injection facility among injection drug users in San Francisco. Drug Alcohol Depend.

[20] O’Rourke A, White RH, Park JN (2019). Acceptability of safe drug consumption spaces among people who inject drugs in rural West Virginia. Harm Reduct J.

[21] Maryland Department of Health. Unintentional drug- and alcohol-related intoxication deaths in Maryland, 2020.; 2021.

[22] Schneider KE, Urquhart GJ, Rouhani S (2021). Practical implications of naloxone knowledge among suburban people who use opioids. Harm Reduct J.

[23] Compton WM, Valentino RJ, DuPont RL (2021). Polysubstance use in the US opioid crisis. Mol Psychiatry.

[24] Karamouzian M, Pilarinos A, Hayashi K, Buxton JA, Kerr T (2022). Latent patterns of polysubstance use among people who use opioids: A systematic review. Int J Drug Policy.

[25] Ciccarone D. The rise of illicit fentanyls, stimulants and the fourth wave of the opioid overdose crisis. Curr Opin Psychiatry. 2021;34(4). https://journals.lww.com/co-psychiatry/Fulltext/2021/07000/The_rise_of_illicit_fentanyls,_stimulants_and_the.4.aspx. 10.1097/YCO.0000000000000717 PMC8154745 33965972

[26] Mattson CL, Tanz LJ, Quinn K, Kariisa M, Patel P, Davis NL (2021). Trends and geographic patterns in drug and synthetic opioid overdose deaths - United States, 2013–2019. MMWR Morb Mortal Wkly Rep.

[27] Strike C, Rudzinski K, Patterson J, Millson M (2012). Frequent food insecurity among injection drug users: correlates and concerns. BMC Public Health.

